# Unilateral Duplicated Collecting System Identified During Pelvic Lymphadenectomy: A Case Report and Literature Review

**DOI:** 10.7759/cureus.26331

**Published:** 2022-06-25

**Authors:** Konstantinos Malligiannis Ntalianis, Candice Cheung, Christina Resta, Sidath Liyanage, Fani Toneva

**Affiliations:** 1 Obstetrics and Gynaecology, Mid & South Essex NHS Foundation Trust, Southend-On-Sea, GBR; 2 Radiology, Gynaecology, and Oncology, Mid & South Essex NHS Foundation Trust, Southend-On-Sea, GBR; 3 Oncology and Gynaecology, Mid & South Essex NHS Foundation Trust, Southend-On-Sea, GBR

**Keywords:** duplex kidney, bifid ureter, anatomic variation, duplex collecting system, double ureter, duplicated ureter

## Abstract

Anatomic variations can be an additional challenge for any surgeon, especially in complicated surgical procedures, and they could lead to complications if not identified timely.

We present a case report of an incidental intraoperative finding of unilateral duplicated ureter, identified during pelvic lymphadenectomy for a patient who underwent surgical treatment for ovarian cancer. Also, we include an update on this anatomic variation following the literature review.

The importance of a systematic approach in the identification of the relevant anatomy and landmarks, taking into consideration the variation of the duplicated ureter, is emphasized in this paper and may contribute beneficially to the experience of surgeons operating in this field.

## Introduction

Ureters are muscle-walled cylindrical tubes with stratified, transitional epithelium which play a significant role in draining urine from the kidney to the bladder in the human body through regular contractions called peristalsis [1]. Typically, ureters are 25-30 cm in length, thick-walled, 3 mm in diameter, and are continuous superiorly with the funnel-shaped renal pelvis. The ureters travel downwards to enter the lateral angle of the urinary bladder. They are classified into two segments, including pelvic and abdominal sections. Abdominal segments are closely linked to the parietal peritoneum and are retroperitoneal through their course, and the pelvic segments enter the pelvis by passing over the pelvic brim at the bifurcation of the common iliac arteries before entering the urinary bladder [2].

Duplicated collecting system is defined as one kidney with two separate renal pelves (duplex kidney) which do not communicate.

The prevalence of unilateral duplicated ureter has been reported at 0.7% to 4%, and the incidence in autopsy studies is 0.5% [3], affecting 2:1 more females than males [4]. The leading cause of duplicated ureter is the abnormality in the ureteric bud branching pattern, which arises from the mesonephric (or Wolffian) duct.

Duplication of the ureter may be complete or incomplete. Incomplete duplication (known as bifid ureter) involves two separate ureters at the proximal aspect, and they join at any point below the uretero-pelvic junction but before entering into the bladder. This happens due to the bifurcation of the ureteric bud prior to its invasion into the metanephric blastema. On the other hand, a ureteral bud that arises twice separately, results in a complete duplication of the ureter (double ureter), which is continuous and leads to two openings into the bladder. The ureter draining from the lower pole of the kidney is the equal to the single-system ureter and hence is called orthotopic, while the upper pole ureter is called ectopic (Weigert-Meyer rule) and is inserted inferior and medial to the orthotopic ureteral orifice [5,6]. In complete duplication, the distal end of the ureter, which comes from the upper moiety, can have an extravesical implantation, such as the vagina, seminal vesicle, urethra, prostate, epididymis, or vas deferens [7]. 

We present a rare case of unilateral duplicated collecting system identified during pelvic lymphadenectomy with a review of the current literature.

Informed written consent form has been obtained by the patient.

## Case presentation

A 67-years-old lady was referred to the suspected cancer pathway of our department for postmenopausal bleeding. From previous medical and gynaecological history, she is parity three, with three previous normal vaginal deliveries and a WHO performance status of zero (0). She had a breast cancer diagnosis more than 15 years ago and was treated with surgery, radiotherapy, and chemotherapy. The patient had regular smear tests, as per the National Cervical Screening programme, which were always normal. Hence, she had no previous large loop excision of the transformation zone (LLETZ) procedures. She is medicated for benign palpitations (secondary to anxiety) and hypercholesterolemia. There was no previous history of any persistent urinary tract symptoms like recurrent infections.

Following the cancer pathway referral, the patient had a transvaginal ultrasound scan of the pelvis, performed by the local primary health care service. On the sonographic examination, the uterus was found to be anteverted of 8 cm size, an endometrial thickness of 10 mm, with a fundal 8 mm calcified fibroid and a 7.2 cm left ovarian complex cyst containing septae, irregular masses, and mural nodular thickening. No free fluid in the pouch of Douglas was seen. The tumour marker of Ca-125 was 24 and hence the risk of malignancy index (RMI) was calculated to be more than 200. Subsequently, an MRI abdomen and pelvis was booked, and the patient was referred to the gynaecological oncology (gynae-oncology) multi-disciplinary team (MDT). A Pipelle endometrial biopsy was obtained, which showed no hyperplasia or malignancy.

**Figure 1:**
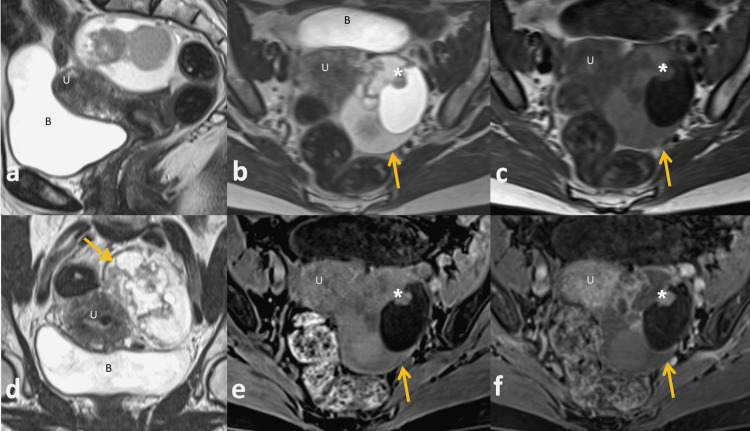
MRI images Sagittal T2 (a), axial T2 (b), axial T1 (c), coronal T2 (d), axial T1 fat-saturation and post-contrast fat-saturation T1 (e-f) . These show a complex multilocular septate left adnexal cyst (arrow) abutting the uterus (U) and posterior to the bladder (B). The cyst contains multiple enhancing septae and an enhancing solid nodule (asterisk). The final pathological diagnosis following surgical excision was an endometrioid adenocarcinoma FIGO stage 1A.

The patient’s case was discussed at the gynae-oncology MDT meeting in view of the whole clinical picture. The MRI demonstrated a left ovarian thin-walled multilocular cystic lesion measuring 80 mm x 60 mm. The cyst was diffusely heterogeneous with different locules containing T2 bright serous fluid and T2 intermediate mucin (Figure 1). There was a large intra-cystic irregular solid component which demonstrates enhancement on the post-contrast sequences. Left adnexal varices were also noted. The overall appearances were highly suspicious for a primary left ovarian malignancy. There were no significantly enlarged para-aortic, pelvic or inguinal nodes, ascites, or discrete peritoneal deposits. The rectum and the urinary bladder appeared normal with no renal pelvicalyceal system dilatation. There was no evidence of extra-pelvic disease.

**Figure 2:**
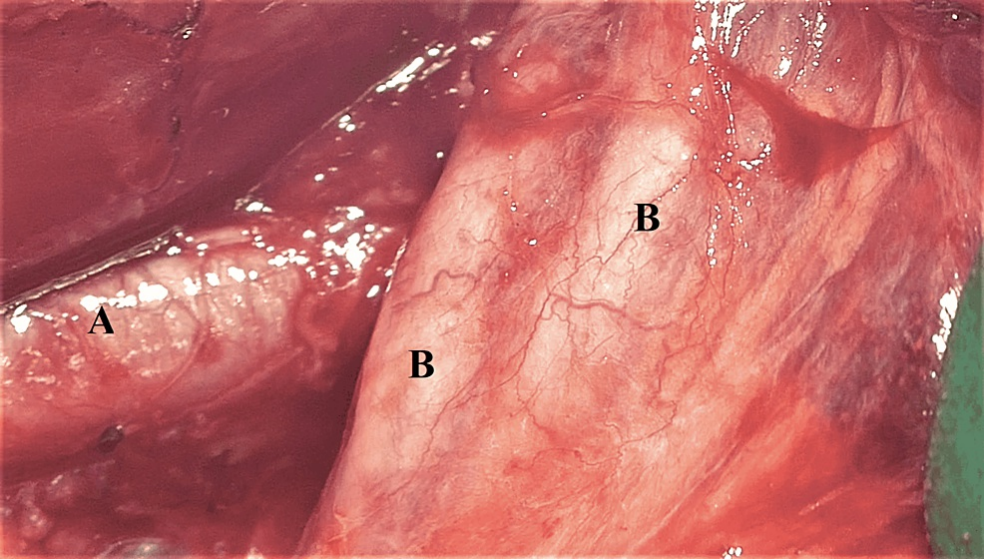
Duplicated ureter identified during pelvic lymphadenectomy on right pelvic sidewall A: right external iliac artery, B: duplicated right ureter

Following the MDT discussion, the patient was consented for total abdominal hysterectomy (TAH), bilateral salpingo-oophorectomy (BSO), frozen section and/or omentectomy, and/or bilateral pelvic lymphadenectomy. The procedure was done under general anaesthesia; she was placed in Lloyd Davis position, cleaned, draped, and catheterized. A midline incision was made to gain access to the intra-abdominal cavity, peritoneal washings were taken for cytology analysis, and a full assessment was done to check for any metastasis. No obvious disease was noted in the abdomen or in the pelvis apart from the known left ovarian cystic lesion. A left salpingo-oophorectomy was performed, and the specimen was sent for frozen section, which suggested endometrioid adenocarcinoma leading to a total omentectomy and bilateral pelvic lymphadenectomy in addition to the TAH and BSO. During the dissection of the right pelvic sidewall, duplicated ureter was seen (Figure 2) as peristalsis confirmed its nature (Video 1). The ureter was mobilised carefully and a yellow sling was placed in order to highlight the ureter and minimize the risk of any injury. Further mobilisation and exposure of the ureters was not done in view of the risk of injury (in an asymptomatic patient) as well as it was not part of the indicated surgical procedure stated on the patient’s consent form. Thus, the type of duplication could not be determined intra-operatively. The left ureter had a normal appearance. 

**Video 1:** Peristalsis of duplicated ureter

The procedure was completed with no complications. Post-operatively, the patient failed her trial without a catheter and had to be re-catheterized due to urine retention and developed subacute bowel obstruction, confirmed by CT abdomen-pelvis with contrast, which was managed conservatively and resolved. Following the urine retention, urine investigations were done showing white blood cells (WBC) 701 x106/L, red blood cells (RBC) 311 x106/L, with no epithelial, hyaline casts, granular casts, nor cellular casts. The culture which followed found >10^5 cfu/ml of Klebsiella pneumoniae sensitive to co-amoxiclav, which was prescribed for one week.

The patient was discharged with a urine catheter in situ and on oral antibiotics. Follow-up appointments were scheduled in one weeks time to be seen by a urology nurse specialist for catheter removal, which was successful, and in four weeks time for surgical follow-up.

The histology confirmed stage 1A/grade 2 left ovarian endometrioid adenocarcinoma with no lymph-vascular space invasion (LVSI). All of the 14 lymph nodes removed were negative. There was no disease within the omentum; the endometrium was found to be proliferative, with a benign endometrial polyp present. The myometrium contained multiple benign leiomyomas, and the cervix showed no evidence of cervical intraepithelial neoplasia (CIN) or cervical glandular intraepithelial neoplasia (CGIN).

On gynae-oncology follow up, the patient had recovered well from the surgery and was completely asymptomatic with no abnormal urinary symptoms. Histology results were explained to the patient as well as the MDT recommendation for only surgical follow-up and no adjuvant treatment.

Considering all of the above facts, with regards to the unilateral duplication of the ureter, this patient had no previous urinary symptoms in her life in order to undergo investigations, which could diagnose earlier this congenital variant. Also, there were no obvious abnormalities in the urinary tract system identified during the pre-operative to raise any suspicion of potential variation of the anatomy. Thankfully, intraoperatively, the surgical team performed a systematic approach in the identification of the landmarks and the anatomy, including the variation, before the dissection of the right sidewall lymph nodes, which prevented any ureteral injury.

**Figure 3:**
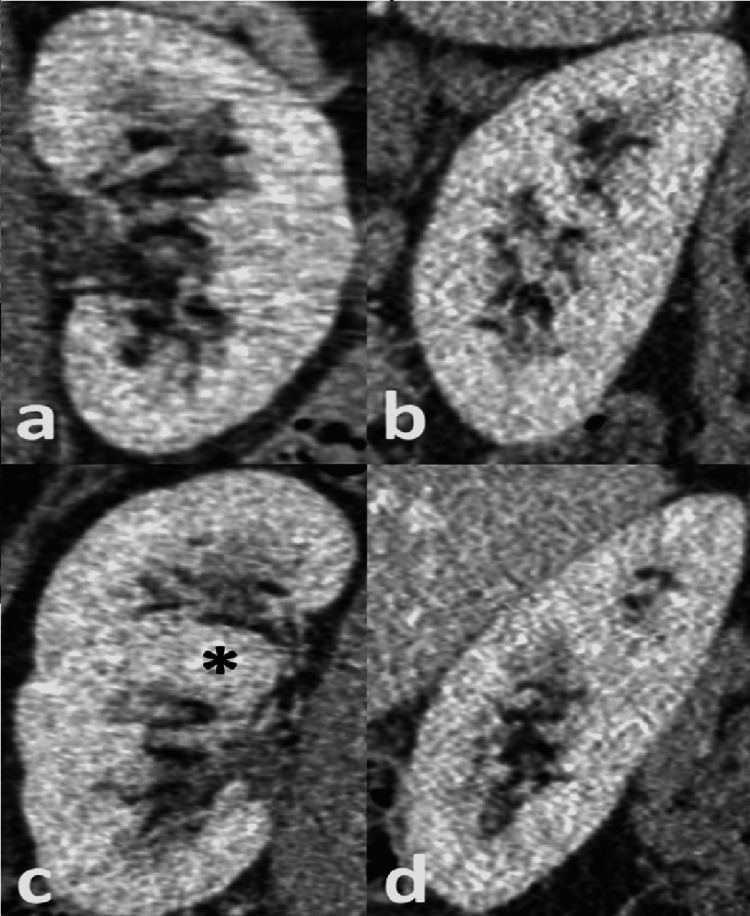
Coronal (a and c) and sagittal (b and d) reformatted images of the left (a and b) and right kidney (c and d) following Iv contrast in the porto-venous phase The right kidney has a duplex appearance with upper and lower moieties separated by a prominent parenchymal bridge (asterisk). In addition, there is asymmetry in the bipolar length measurements, right kidney = 11cm and left kidney = 9 cm, a common finding in duplex kidney. The ureters are not clearly identified on these images, which usually requires a delayed excretory phase, not performed in this case.

A retrospective review of the available pre-operative MRI abdomen-pelvis and post-operative CT abdomen-pelvis with contrast could not result in a safe conclusion of the type of duplication in this case. The only finding, which was noted during the retrospective review of the post-operative CT abdomen-pelvis was the right duplex kidney (Figure 3). However, there was no excretory phase with contrast in the collecting system in order to visualize clearly the ureter.

## Discussion

The ureter develops during the fifth week of embryological development. The ureteric bud arises as a diverticulum from the caudal end of the mesonephric (Wolffian) duct and invades the metanephric blastema [8]. The glial cell-derived neurotrophic factor (GDNF)-RET is suggested to regulate the induction and branching of ureteric bud [9]. This fusion forms the renal pelvis, which on division gives rise to major and minor calyces (parenchyma). Therefore, the ureteric bud gives origin to the collecting system, including the ureter, pelvis, major and minor calyces, and metanephric blastema forms the kidney, that is, glomerulus, capsule, and nephron tubules. Disruptions in the embryologic development can cause congenital abnormalities of the kidney and urinary tract.

Congenital anomalies of urinary tracts, double ureter, and kidneys comprise 20% to 30% of prenatal anomalies [1]. Ureteric variations include (i) variations in number (single ureter, double, triple, etc.), (ii) complete, incomplete, or branching, and (iii) variations in morphology (hydroureter, imperforate ureter, diverticulum of ureter, ectopic ureter). However, the main variation of the ureter is the division into two [3].

From data derived from autopsy studies, it has been suggested that the unilateral bifid ureter’s incidence is found 1 in 125 cases (0.8%) [2]. Kasat et al. have reported two cases (2.2%), both males, with unilateral incomplete duplicated ureters in a study of 90 adult human cadavers [10]. Also, in another cadaveric study, Choudhary et al. analyzed 32 specimens and determined that two (6.25%) kidneys showed unilateral incomplete duplication [11]. Another recent study demonstrated incomplete duplicated ureter in three (6%) of 50 specimens, one on the left side and two on the right side. Compared to other studies, this study showed a relatively high prevalence of double ureter among various studies, which may indicate a higher prevalence of duplex collecting systems in the South Indian population. Additionally, it is assumed that duplicated ureters are unilateral, affecting one side of the kidney [7]. Regarding the complete duplication, Deka and Saika experimented on 60 specimens and found that 1.67% of the models had double ureters on the left side [12]. Another study by Roy et al., where they evaluated 156 kidney specimens, quoted a rate of 0.64% with double ureters [13]. Siomou et al., in a retrospective study evaluating children with urinary tract infection, explained that duplicated ureters are affecting more the females than males by 2:1, equipped with unilateral presentation (81% of the cases), and complete duplication was as common as the incomplete duplication [14].

The majority of patients with duplicated ureter remain asymptomatic, but some complications can be persistent. The vast majority of these cases are diagnosed in early life with recurrent urinary tract infections [14]. In adult life, patients can present with haematuria, abdominal or flank pain, and predisposition to obstruction, vesicoureteral reflux, and recurrent urinary infections depending on the type of the duplication [6]. Complete duplication is associated with the ectopic ureterocele, vesicoureteral reflux, and ectopic ureteral insertion. Incomplete duplication is linked to the ureteropelvic junction or ureteroureteral reflux obstruction of the kidney lower pole [15].

Additionally, the congenital duplex collecting system can potentially increase the risk of ureteral injury during surgery. Iatrogenic ureteral injuries are not a rare complication of any open or laparoscopic surgical procedure involving the pelvis, especially in gynaecological procedures [4]. The overall incidence of intraoperative, iatrogenic ureteral injury has been reported between 0.5 to 10% [16].

Preoperative ureteral radiographic imaging by intravenous urography or computed tomography (CT) has been highlighted in cases of large pelvic masses, pelvic inflammatory disease, or endometriosis. Furthermore, ureteric stenting could facilitate the identification of anatomy in the surgical field in complex cases or in cases suspected of congenital variations/anomalies, minimizing iatrogenic ureteric injury. However, it is not recommended on a routine basis. [17].

The diagnosis and follow-up assessments of ureteric duplication can be performed usually with ultrasonography, intravenous pyelography (IVP), voiding cystourethrography (VCUG), CT, and magnetic resonance urography (MRU). In general, ultrasonography is considered to be the first-line imaging modality as it can demonstrate findings suggestive of duplication, such as asymmetrical renal lengths, separated renal pelvises, or disproportionate, pelvicalyceal dilation of the upper and lower poles, presence of ureterocele, especially in females, where it has a stronger association with duplicated collecting system. Also, VCUG is the standard imaging method performed in the assessment for vesicoureteral reflux, which is common in duplicated collecting system cases. IVP has been used to assess the morphology and function of the urinary tract. Following intravenous administration of contrast agent, the appearance of the kidneys, ureters, and bladder are examined for obstruction, scarring, or other abnormalities by radiographs and fluoroscopy. Furthermore, comparing MRU with CT, the spatial resolution of MRU is less than that of CT; however, the contrast resolution is superior and hence the urine in the collecting system acts as a contrast agent in MRU. The main findings of duplicated renal collecting systems are the same as on CT as they were described above in ultrasonography. On the other hand, CT reconstruction software can generate very good images of the collecting systems [18]. Overall, each imaging modality can contribute to a better understanding of the effect of the duplex anomaly [5]. 

Finally, with regards to the treatment, it should be guided by the symptoms secondary to diagnosis as well as by the renal function. In the study of Siomou et al., out of the 75 duplicated collecting systems, 24 were managed surgically (19 with complete duplication and five with incomplete). Of the 40 duplex systems which were complicated with vesicoureteral reflux, 26 were managed conservatively with the prophylactic use of antibiotics [14]. During, the follow-up resolution of reflux tended to be higher in incomplete duplication than complete (seven to three cases). To conclude, suspicion or diagnosis of duplicated collecting system in a patient, especially in children, is an indication for pediatric urologist referral or MDT input.

## Conclusions

The importance of a systematic approach in the identification of the relevant anatomy and landmarks, taking into consideration the variation of the duplicated collecting system, is highlighted in this case report providing an insight into this congenital anomaly not only to the surgeons who are operating in the field of the lesser pelvis but also to radiologists whose role is crucial in the multi-disciplinary team input to each case.
